# Artificial intelligence policies in neurology journals: a cross-sectional analysis

**DOI:** 10.3389/fneur.2026.1766696

**Published:** 2026-02-17

**Authors:** Kohl Kirby, Noah Calvert, Kaylyn Rowsey, Jillian Brassfield, Taylor Gardner, Patrick Crotty, Alec Young, Andrew Tran, Alicia Ito Ford, Matt Vassar

**Affiliations:** 1 Office of Student Medical Research, Oklahoma State University Center for Health Sciences, Tulsa, OK, United States; 2 Department of Psychiatry and Behavioral Sciences, Oklahoma State University Center for Health Sciences, Tulsa, OK, United States

**Keywords:** artificial intelligence, neurology–clinical, reporting guideline, research ethics, research integrity

## Abstract

**Introduction:**

Artificial intelligence (AI) is increasingly used in neurology research and scientific publishing. However, concerns regarding authorship, transparency, and ethical oversight have prompted journals to establish policies governing AI use. The objective of this study was to characterize the presence and content of AI-related author-guideline policies across the top 100 neurology journals and to evaluate their alignment with established editorial frameworks and AI-specific reporting guidelines.

**Methods:**

We conducted a cross-sectional analysis of the top 100 neurology journals. Data were extracted from each journal’s Instructions for Authors and included policies regarding AI use, disclosure requirements, authorship restrictions, and permissions for AI-assisted writing, AI-generated content, and AI-generated images. References to ethical frameworks and AI-specific reporting guidelines were also recorded. Associations between AI policies and journal metrics were assessed.

**Results:**

Of the 100 journals examined, 97 included an AI-related policy. Nearly all journals prohibited AI authorship (97%) and required disclosure of AI use (96%). AI-assisted writing was widely permitted (93%), whereas permissions for AI-generated content (77%) and AI-generated images (37%) were more variable. Endorsement of AI-specific reporting guidelines was rare, with only one journal referencing CONSORT-AI or SPIRIT-AI. Few journals cited established ethical frameworks, including the International Committee of Medical Journal Editors (ICMJE; 14%), the Committee on Publication Ethics (COPE; 26%), and the World Association of Medical Editors (WAME; 10%). No significant correlations were identified between AI-related policies and journal metrics.

**Discussion:**

AI-related policies are common among neurology journals but remain heterogeneous and inconsistently aligned with established ethical and methodological standards. These findings highlight opportunities to strengthen transparency and research integrity as AI becomes increasingly integrated into neurological science. Neurology journals should consider adopting standardized requirements for AI-use disclosure and explicitly endorsing AI-specific reporting frameworks to harmonize expectations and improve reproducibility.

## Introduction

Artificial intelligence (AI) is rapidly becoming a transformative tool in neurology, with expanding applications across clinical practice and research. In clinical settings, AI has been used to support diagnostic and decision-making processes, including applications in neuroimaging and electrophysiology (1–4). This growth in clinical interest is mirrored in research, where AI has facilitated large-scale data analysis and improved understanding of disease mechanisms and treatment efficacy, including in neuropathology and neurodegenerative diseases such as Alzheimer’s and Parkinson’s (5–13). In addition, natural language processing and automated data extraction tools have improved the efficiency of systematic reviews, meta-analyses, and clinical studies (14). While these advances offer substantial opportunities, they also introduce challenges that may undermine the quality, transparency, and integrity of scientific work.

A major concern is the risk of bias in AI models, which rely on training data. If datasets lack diversity, models may produce skewed or discriminatory results (15). In neurology, where diseases affect diverse populations, biased AI can perpetuate disparities in diagnosis, treatment, and research, potentially leading to misinterpretation or missed diagnoses when applied to underrepresented groups (16). Additionally, the “black box” nature of many AI algorithms complicates their use in research. The lack of transparency makes it difficult for researchers to understand how AI systems arrive at conclusions, undermining trust in findings and impeding the reproducibility of results (17). In fields like neurology, where precision is critical, this opacity can jeopardize scientific rigor (18). When research outcomes depend on tools that cannot be fully understood or explained, the integrity of the research itself is at risk.

As the role of AI continues to expand in neurology research, the growing reliance on these technologies raises important ethical and practical concerns. With AI analyzing vast amounts of data and assisting in manuscript preparation, there is a risk that poorly validated AI models may produce false or misleading results. If published, these inaccuracies could have significant consequences for both clinical practice and future research (19). Furthermore, AI-assisted writing tools, while efficient, may generate drafts that lack the clarity or depth of analysis needed for high-quality scientific communication, ultimately compromising the quality of published research (20).

Given the rapid adoption of AI in neurology research, there is a clear need for regulation and oversight to address these challenges. Without such regulation, the promise of AI to advance neurology research could be overshadowed by the risks it introduces to data quality, research accuracy, and scientific rigor. This study seeks to evaluate how neurology journals are addressing the opportunities and challenges posed by AI in research. Through a systematic review of the author guidelines from leading neurology journals, we examine the extent to which these journals have established policies on AI usage. Specifically, we focus on transparency standards, ethical considerations, and protocols for reporting AI-driven contributions to research. This work fills a critical gap in understanding how well neurology journals are equipped to handle the complexities of AI integration—an issue that is becoming increasingly important. By highlighting current practices and identifying gaps in these guidelines, our study provides valuable insights for refining journal policies, encouraging responsible AI usage, and maintaining the integrity of neurology research in the age of technological innovation.

## Methods

### Study design

We conducted a cross-sectional analysis of the manuscript submission guidelines for the top 100 journals, as ranked by the 2023 SCImago Journal Rankings. Data were collected from the Instructions for Authors webpages of these 100 journals. This study adhered to the Strengthening the Reporting of Observational Studies in Epidemiology (STROBE) guidelines.

### Search strategy

The selection of eligible journals was carried out through consultation between the two investigators (PC, AVT) and the medical research librarian. The 2023 SCImago Journal Rankings were used to gather 396 journal listings for screening. SCImago is an online platform that ranks journals using bibliometric indicators, including the h-index and the SCImago Journal Rank Indicator (SJR). The SJR is considered a more robust metric than the traditional impact factor, as it is open-access and evaluates both the quantity and quality of a publication’s citations. The rankings are generated annually using Elsevier’s subscription-based Scopus database, which encompasses a vast collection of scientific journals, providing a comprehensive dataset for this study. All peer-reviewed journals categorized under subject area ‘medicine’ and subject category ‘neurology’ of the 2023 SCImago journal listings were included in this study.

### Exclusion criteria

Journals were excluded from our study if they met any of the following criteria: (i) were discontinued, (ii) lacked editorial office contact information on their website, as we sought to minimize bias by providing editors an opportunity to clarify their publication policies, or (iii) were published in a language other than English without offering a translation option.

### Data extraction process

Data was independently extracted by two investigators (KK, NC) from the Instructions for Authors pages of included journals in a masked, duplicate manner. The data were recorded using a standardized Google Form, which was pilot-tested and designed in advance by investigators PC and AVT. During this process, we carefully reviewed publishing policies, authorship criteria, and any editorials or updates from journals and publishing companies related to the use of AI, chatbots, and large language models (LLMs). Data extraction occurred between November 1, 2024 and July 31, 2025. Once the extraction was completed, investigators KK and NC reconciled their data, with a third investigator (PC) available to resolve any discrepancies. All journals were re-reviewed prior to final data reconciliation to ensure that policy information was accurate as of July 31, 2025.

### Editorial outreach

For journals without a statement on AI usage in their Instructions for Authors webpage, a standardized email was sent to the Editor-in-Chief or a member of the editorial office to inquire about the development of policies regarding AI use in their publication process. To enhance response rates, emails were sent weekly for three consecutive weeks. All responses, including non-responses, were documented.

### Outcomes

The primary outcome of this study evaluated AI usage statements in journal guidelines. The secondary outcome involved reviewing and summarizing the number of journals that permit or prohibit the use of AI-generated content, images, writing, and the inclusion of AI authorship.

### Data synthesis

Data summaries of descriptive statistics for journal AI policies were created using R (version 4.2.1) and RStudio. These descriptive statistics consisted of statements regarding: (1) AI generated images (2) AI generated content (3) AI authorship inclusion (4) AI assisted manuscript writing. Bias analysis was not necessary, as the data collected involved a direct evaluation of the Instructions for Authors as opposed to an assessment of each individual study.

In addition, R (version 4.2.1) and RStudio were used to conduct correlational analyses. The strength of correlation was analyzed between the AI usage policies in research and SJR score, journal rank, impact factor, and publishing country. Point-biserial correlations were used because AI policy variables were dichotomous (e.g., permitted vs. not permitted), whereas journal metrics were continuous, making point-biserial correlation an appropriate effect-size measure equivalent to Pearson correlation in this setting. Given the exploratory nature of these analyses, we considered multiple-comparison adjustment but did not apply formal correction; exact *p*-values are reported for transparency.

### Reproducibility

To ensure transparency and reproducibility, analysis scripts, standardized emails, extraction forms, and all raw data collected are publicly available on Open Science Framework (OSF) (21). Generative AI (ChatGPT, OpenAI; GPT-5.1) was used solely to assist with grammar and wording refinement during manuscript preparation; it was not used for data extraction, analysis, interpretation, or content generation, and all edits were reviewed for accuracy by the authors.

## Results

**Figure 1:**
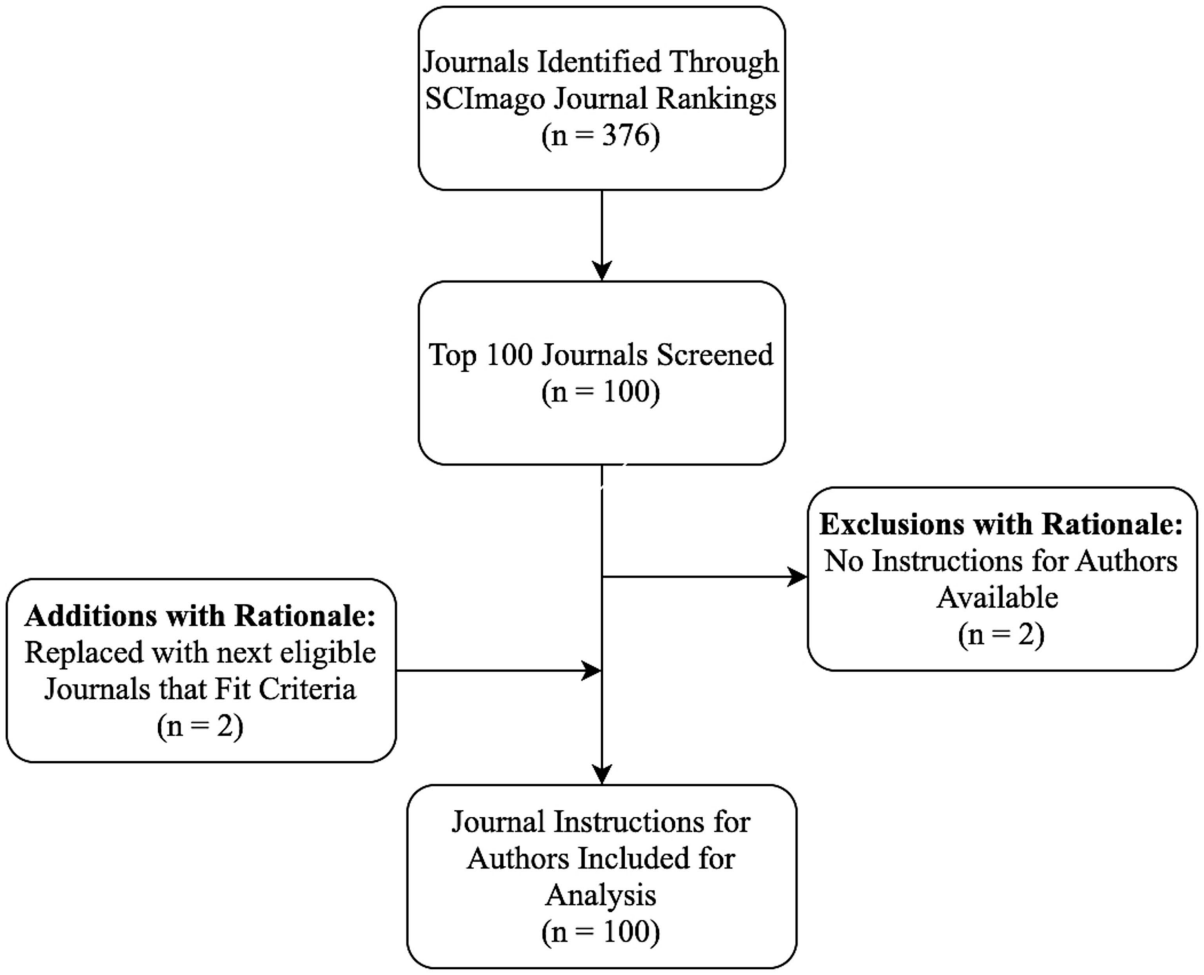
Flow diagram for study selection. This diagram illustrates the identification, screening, and inclusion process for neurology journals assessed in the study. A total of 376 journals were initially identified through the 2023 SCImago Journal Rankings. The top 100 journals were screened, after which two journals were excluded due to the absence of an available Instructions for Authors. These were replaced with two journals that met inclusion criteria, resulting in 100 journals included for analysis.

A total of 100 neurology journals were included in the analysis following screening and replacement of two journals that lacked Instructions for Authors (Figure 1). Journal characteristics are summarized in Table 1. Nearly half were published in North America (49/100, 49.0%) and a similar proportion in Europe (47/100, 47.0%). Most journals were ranked in the first SJR quartile (97/100, 97.0%). The median SCImago Rank was 52 (IQR 27–77), and the median 2023 Journal Impact Factor was 4.5 (IQR 3.6–6.7). Springer Nature (18/100, 18.0%), Wiley-Blackwell (15/100, 15.0%), and Elsevier (14/100, 14.0%) were the most frequently represented publishers.

**Table 1:** Journal characteristics.

Characteristic	*N* = 100
Continent, n (%)	
North America	49 (49.0)
Europe	47 (47.0)
Asia	4 (4.0)
SJR Quartile, n (%)	
Q1	97 (97.0)
Q2	3 (3.0)
SCImago Rank, Median (IQR)	52 (27–77)
2023 Journal Impact Factor, Median (IQR)	4.5 (3.6–6.7)
Publisher, n (%)	
Springer Nature	18 (18.0)
Wiley-Blackwell	15 (15.0)
Elsevier	14 (14.0)
SAGE Publications	12 (12.0)
Oxford University Press	7 (7.0)
Lippincott Williams and Wilkins	6 (6.0)
BMJ Group	4 (4.0)
American Association of Neurological Surgeons	3 (3.0)
IOS Press	2 (2.0)
Karger	2 (2.0)
Taylor and Francis	2 (2.0)
Adis	1 (1.0)
American Academy of Sleep Medicine	1 (1.0)
American Heart Association	1 (1.0)
American Medical Association	1 (1.0)
American Society of Neuroradiology	1 (1.0)
Annual Reviews	1 (1.0)
Bentham Science	1 (1.0)
Cambridge University Press	1 (1.0)
Current Medicine Group	1 (1.0)
International Society on Aging and Disease	1 (1.0)
Korean Spinal Neurosurgery Society	1 (1.0)
Korean Stroke Society	1 (1.0)
Masson	1 (1.0)
Portland Press	1 (1.0)
Wolters Kluwer Medknow	1 (1.0)

Journal characteristics of the top 100 neurology journals. This table summarizes descriptive characteristics of the 100 neurology journals included in the analysis, including geographic distribution, SCImago Journal Rank (SJR) quartile, median SCImago Rank, 2023 Journal Impact Factor, and publishing organization.

### General editorial guidelines

Most journals (97/100, 97.0%) referenced artificial intelligence within their Instructions for Authors (Table 2). Fewer endorsed established editorial frameworks: 14.0% (14/100) cited International Committee of Medical Journal Editors (ICMJE) recommendations, 26.0% (26/100) cited Committee on Publication Ethics (COPE) guidance, and 10.0% (10/100) referenced World Association of Medical Editors (WAME). Only one journal (1/100, 1.0%) endorsed an AI-specific reporting guideline. The three journals without an AI statement were contacted via editorial outreach; no responses were received and no policy classifications changed.

**Table 2:** General journal guidelines.

Characteristic	*N* = 100
AI mentioned in the Instructions for Authors, n (%)	
Yes	97 (97.0)
No	3 (3.0)
ICMJE Statement, n (%)	
Yes	14 (14.0)
No	86 (86.0)
COPE Statement, n (%)	
Yes	26 (26.0)
No	74 (74.0)
WAME Statement, n (%)	
Yes	10 (10.0)
No	90 (90.0)
AI Specific Reporting Guideline, n (%)	
Yes	1 (1.0)
No	99 (99.0)
Journal recommend or require adherence to said guideline(s), n (%)	
NA	99 (99.0)
Required	1 (1.0)
Specific Guidelines, n (%)	
CONSORT-AI, SPIRIT-AI	1 (100.0)
Unknown	99

General editorial guideline characteristics across neurology journals. This table presents the frequency of journals reporting AI-related statements within their Instructions for Authors. It includes endorsement of ICMJE, COPE, and WAME guidance, as well as adoption of AI-specific reporting guidelines such as CONSORT-AI and SPIRIT-AI.

### AI-related publication policies among all journals

AI authorship was universally prohibited among journals with clear policies, with 97.0% (97/100) explicitly disallowing AI tools as authors (Table 3). Disclosure of AI use during manuscript submission was required by 96.0% (96/100) of journals. AI-assisted writing was widely permitted (93/100, 93.0%). Policies regarding AI-generated outputs were more variable: 77.0% (77/100) allowed AI-generated content, whereas 37.0% (37/100) allowed AI-generated images.

**Table 3:** AI guidelines.

Characteristic	*N* = 100
AI tools allowed for Authorship, n (%)	
Yes	0 (0.0)
No	97 (97.0)
Not Stated	3 (3.0)
Require authors to disclose the use of AI during submission, n (%)	
Yes	96 (96.0)
No	1 (1.0)
Not Stated	3 (3.0)
AI tools allowed in Manuscript Writing, n (%)	
Yes	93 (93.0)
No	0 (0.0)
Not Stated	7 (7.0)
AI tools allowed in content generation, n (%)	
Yes	77 (77.0)
No	16 (16.0)
Not Stated	7 (7.0)
AI tools allowed in image generation, n (%)	
Yes	37 (37.0)
No	39 (39.0)
Not Stated	24 (24.0)

AI-related publication policies among all included neurology journals. This table reports the frequency of journals permitting or prohibiting various forms of AI use, including authorship, manuscript writing assistance, content generation, image generation, and disclosure requirements for AI use during submission.

### AI-related publication policies among journals explicitly mentioning AI

Among journals that explicitly referenced AI in their guidelines (*n* = 97), all prohibited AI authorship (97/97, 100%) and nearly all required disclosure of AI use (96/97, 99.0%; Table 4). AI-assisted writing was permitted by 95.9% (93/97). Policies on AI-generated content (77/97, 79.4%) and AI-generated images (37/97, 38.1%) remained heterogeneous.

**Table 4:** AI guidelines in journals mentioning AI.

Characteristic	*N* = 97
AI tools allowed for Authorship, n (%)	
Yes	0 (0.0)
No	97 (100.0)
Not Stated	0 (0.0)
Require authors to disclose the use of AI during submission, n (%)	
Yes	96 (99.0)
No	1 (1.0)
Not Stated	0 (0.0)
AI tools allowed in Manuscript Writing, n (%)	
Yes	93 (95.9)
No	0 (0.0)
Not Stated	4 (4.1)
AI tools allowed in content generation, n (%)	
Yes	77 (79.4)
No	16 (16.5)
Not Stated	4 (4.1)
AI tools allowed in image generation, n (%)	
Yes	37 (38.1)
No	39 (40.2)
Not Stated	21 (21.6)

AI-related publication policies among journals explicitly mentioning AI. This table summarizes AI-related editorial policies for the subset of journals that reference AI in their author guidelines, including permissions or restrictions on AI authorship, writing assistance, content generation, image generation, and disclosure requirements.

### Biserial correlations

No significant associations were observed between AI-related policies and journal characteristics, including Impact Factor, SCImago Rank, or publisher. Prespecified correlation analyses are reported in Supplementary Additional File 1.

## Discussion

Our study provides a comprehensive evaluation of how leading neurology journals are addressing the rapid integration of AI into scientific research and publishing. By systematically analyzing the Instructions for Authors of the top 100 neurology journals, we identified substantial inconsistencies in AI-related policies, underscoring the lack of standardization across the field. Although nearly all journals acknowledged AI in their submission guidelines and mandated disclosure of AI use, their policies varied widely in scope, specificity, and enforcement. These findings highlight both the growing recognition of AI’s impact in research and the urgent need for clearer frameworks to ensure its responsible use (22). Notably, we observed no meaningful associations between AI policy adoption and journal metrics, suggesting that implementation may reflect publisher-level governance or ethical positioning rather than citation-based prestige.

### Variability and gaps in journal AI policies

The majority of neurology journals in our study prohibited AI authorship and required disclosure of AI use, aligning with the recommendations of the ICMJE (23). However, notable heterogeneity emerged regarding which forms of AI assistance were permissible. While nearly all journals (95.9%) allowed AI for language refinement or writing assistance, only 79.4% permitted AI in content generation and less than half (38.1%) endorsed AI-generated images. This variability reflects an ongoing tension between encouraging technological innovation and maintaining the integrity of scientific communication (24). Such inconsistencies are not unique to neurology. In ophthalmology, for example, Almobayed et al. reported that only two-thirds of journals had implemented AI policies, a finding that mirrors the uneven adoption we observed (25). Similar discrepancies across disciplines indicate that policy development is lagging behind technological progress, creating uncertainty for authors and editors alike (26).

### Importance of AI-specific reporting guidelines

Despite the ubiquity of AI in contemporary research, adherence to AI-specific reporting guidelines was almost nonexistent in our sample, only one journal referenced the Consolidated Standards of Reporting Trials–Artificial Intelligence (CONSORT-AI) and Standard Protocol Items: Recommendations for Interventional Trials–Artificial Intelligence (SPIRIT-AI) extensions (27, 28). These guidelines were designed to enhance transparency, reproducibility, and interpretability in studies that develop or evaluate AI interventions (29). Their absence in neurology journals raises concerns about the reporting quality of AI-driven studies. Even when broader editorial and ethical frameworks were considered, adherence remained limited. Only 14% of journals referenced ICMJE, 26% cited COPE, and 10% mentioned WAME in regards to their AI policies (23, 30, 31). These figures indicate that, while some recognition of established ethical guidance exists, consistent enforcement across neurology journals is lacking. Each of these organizations has recently begun addressing AI’s role in scientific writing, uniformly prohibiting AI authorship, mandating disclosure of AI assistance, and affirming that human authors bear full responsibility for verifying the accuracy and integrity of all AI-generated content (32). Integrating these limited ethical frameworks with robust, AI-specific reporting guidelines would provide a more cohesive and enforceable foundation for responsible AI use across both research conduct and manuscript preparation (22).

### The role and benefits of AI in research

While the current lack of standardization presents challenges, AI also offers substantial benefits to neurological science. AI-assisted data analysis can accelerate hypothesis generation, detect subtle imaging biomarkers, and uncover complex patterns in high-dimensional datasets that exceed human analytic capacity (33–36). Natural language processing tools have demonstrated efficiency in screening abstracts and extracting data for systematic reviews, thereby reducing researcher workload and enhancing reproducibility (37, 38). However, these advantages are contingent upon transparent documentation of AI involvement. Unregulated or undisclosed use risks propagating bias or producing misleading conclusions, which could ultimately compromise clinical translation (39). Therefore, enforcing standardized policies is not merely procedural, it is essential for maintaining scientific rigor as neurology increasingly embraces computational methodologies.

### Strengths and limitations

This study possesses several strengths, especially in its design. To minimize bias and improve accuracy, data extraction was performed in a masked, duplicate manner with two trained authors. Additionally, we adhered to a pre-established protocol, ensuring a structured and systematic approach. While this study has many strengths, it is not without limitations. Human error in data extraction and analysis remains a possibility; however, we aimed to minimize this risk through reconciliation, discussion, and the involvement of a third party to resolve any discrepancies. Additionally, some neurology journals lacked standardized Instructions for Authors, which may have led to missed information if AI policies were only accessible through external links. To address this, we contacted editorial teams to verify the existence of an AI policy when it was initially coded as absent.

### Relevance of AI guidelines in neurology

The ethical and methodological stakes of AI integration are particularly high in neurology, where diagnostic accuracy and interpretive precision are paramount (40). To meet these demands, AI systems require sufficient transparency to allow clinicians and reviewers to evaluate how predictions are derived and whether they align with neurobiological principles. Standardized reporting guidelines, such as CONSORT-AI and SPIRIT-AI, could be implemented to help mitigate these risks by enforcing transparency in dataset composition, model validation, and interpretability (27, 28). Furthermore, providing clear guidance on the permissible use of generative AI in writing and figure creation would ensure that human oversight remains central to scientific authorship (41). As AI continues to shape the landscape of neurological research, journals must act proactively to establish enforceable, transparent, and ethically sound frameworks.

## Conclusion

Our analysis reveals that while most neurology journals have acknowledged the growing role of AI in research, their policies remain fragmented, inconsistently applied, and often lack reference to established reporting standards. To preserve the credibility and rigor of neurology research, journals and publishers should collaborate to implement uniform, enforceable policies that promote transparency and ethical responsibility in AI use. Encouraging adoption of AI-specific reporting frameworks and harmonizing disclosure requirements across publishers will be essential to ensuring that the integration of AI strengthens rather than undermines the scientific enterprise.

## Data Availability

The datasets presented in this study can be found in online repositories. The names of the repository/repositories and accession number(s) can be found at: https://osf.io/dc23r/?view_only=cc0f111783d7489090498a4982584251.

## References

[1] Voigtlaender S Pawelczyk J Geiger M Vaios EJ Karschnia P Cudkowicz M . Artificial intelligence in neurology: opportunities, challenges, and policy implications. J Neurol. (2024) 271:2258–73. doi: 10.1007/s00415-024-12220-8, 38367046 PMC12239762

[2] Hillis JM Bizzo BC. Use of artificial intelligence in clinical neurology. Semin Neurol. (2022) 42:39–47. doi: 10.1055/s-0041-1742180 35576929

[3] Bonkhoff AK Grefkes C. Precision medicine in stroke: towards personalized outcome predictions using artificial intelligence. Brain. (2022) 145:457–75. doi: 10.1093/brain/awab439, 34918041 PMC9014757

[4] Smith CM Weathers AL Lewis SL. An overview of clinical machine learning applications in neurology. J Neurol Sci. (2023) 455:122799. doi: 10.1016/j.jns.2023.122799 37979413

[5] Marzi SJ Schilder BM Nott A Frigerio CS Willaime-Morawek S Bucholc M . Artificial intelligence for neurodegenerative experimental models. Alzheimers Dement. (2023) 19:5970–87. doi: 10.1002/alz.13479, 37768001

[6] Jin L Shi F Chun Q Chen H Ma Y Wu S . Artificial intelligence neuropathologist for glioma classification using deep learning on hematoxylin and eosin stained slide images and molecular markers. Neuro-Oncology. (2021) 23:44–52. doi: 10.1093/neuonc/noaa163, 32663285 PMC7850049

[7] Komori T. AI neuropathologist: an innovative technology enabling a faultless pathological diagnosis? Neuro-Oncology. (2021) 23:1–2. doi: 10.1093/neuonc/noaa229, 33059363 PMC7850128

[8] Signaevsky M Prastawa M Farrell K. Artificial intelligence in neuropathology: deep learning-based assessment of tauopathy. Lab Investig. (2019) 99:1019–29. doi: 10.1038/s41374-019-0202-4 30770886 PMC7684013

[9] Koga S Ikeda A Dickson DW. Deep learning-based model for diagnosing Alzheimer’s disease and tauopathies. Neuropathol Appl Neurobiol. (2022) 48:e12759. doi: 10.1111/nan.12759, 34402107 PMC9293025

[10] Koga S Zhou X Dickson DW. Machine learning-based decision tree classifier for the diagnosis of progressive supranuclear palsy and corticobasal degeneration. Neuropathol Appl Neurobiol. (2021) 47:931–41. doi: 10.1111/nan.12710, 33763863 PMC9292481

[11] Koga S Ghayal NB Dickson DW. Deep learning-based image classification in differentiating tufted astrocytes, astrocytic plaques, and neuritic plaques. J Neuropathol Exp Neurol. (2021) 80:306–12. doi: 10.1093/jnen/nlab005, 33570124 PMC7985829

[12] Shakir MN Dugger BN. Advances in deep neuropathological phenotyping of Alzheimer disease: past, present, and future. J Neuropathol Exp Neurol. (2022) 81:2–15. doi: 10.1093/jnen/nlab122, 34981115 PMC8825756

[13] Wong DR Tang Z Mew NC Das S Athey J McAleese KE . Deep learning from multiple experts improves identification of amyloid neuropathologies. Acta Neuropathol Commun. (2022) 10:66. doi: 10.1186/s40478-022-01365-0, 35484610 PMC9052651

[14] Jiang F Jiang Y Zhi H Dong Y Li H Ma S . Artificial intelligence in healthcare: past, present and future. Stroke Vasc Neurol. (2017) 2:230–43. doi: 10.1136/svn-2017-000101, 29507784 PMC5829945

[15] Norori N Hu Q Aellen FM Faraci FD Tzovara A. Addressing bias in big data and AI for health care: a call for open science. Patterns (N Y). (2021) 2:100347. doi: 10.1016/j.patter.2021.100347, 34693373 PMC8515002

[16] Paul S Maindarkar M Saxena S Saba L Turk M Kalra M . Bias investigation in artificial intelligence systems for early detection of Parkinson’s disease: a narrative review. Diagnostics. (2022) 12:166. doi: 10.3390/diagnostics12010166, 35054333 PMC8774851

[17] Shortliffe EH Sepúlveda MJ. Clinical decision support in the era of artificial intelligence. JAMA. (2018) 320:2199–200. doi: 10.1001/jama.2018.17163, 30398550

[18] Cabitza F Rasoini R Gensini GF. Unintended consequences of machine learning in medicine. JAMA. (2017) 318:517–8. doi: 10.1001/jama.2017.7797, 28727867

[19] Taloni A Scorcia V Giannaccare G. Large language model advanced data analysis abuse to create a fake data set in medical research. JAMA Ophthalmol. (2023) 141:1174–5. doi: 10.1001/jamaophthalmol.2023.5162, 37943569 PMC10636646

[20] Khlaif ZN Mousa A Hattab MK. The potential and concerns of using AI in scientific research: ChatGPT performance evaluation. JMIR Med Educ. (2023) 9:e47049. doi: 10.2196/47049 37707884 PMC10636627

[21] Tran AV Young A Crotty P Vassar M. 2024–2025 Winter projects: Endorsement of artificial intelligence guidelines across specialty medical journals: A series of cross-sectional reviews OSF (2024).

[22] Kolbinger FR Veldhuizen GP Zhu J Truhn D Kather JN. Reporting guidelines in medical artificial intelligence: a systematic review and meta-analysis. Commun Med. (2024) 4:71. doi: 10.1038/s43856-024-00492-0, 38605106 PMC11009315

[23] International Committee of Medical Journal Editors (ICMJE). Available online at: https://www.icmje.org/recommendations/browse/roles-and-responsibilities/defining-the-role-of-authors-and-contributors.html (Accessed March 8, 2025).

[24] BaHammam AS. Balancing innovation and integrity: the role of AI in research and scientific writing. Nat Sci Sleep. (2023) 15:1153–6. doi: 10.2147/NSS.S455765, 38170140 PMC10759812

[25] Almobayed A Eleiwa TK Badla O Khodor A Ruiz-Lozano RE Elhusseiny AM. Do ophthalmology journals have AI policies for manuscript writing? Am J Ophthalmol. (2025) 271:38–42. doi: 10.1016/j.ajo.2024.11.003, 39515455

[26] Bhavsar D Duffy L Jo H Lokker C Haynes RB Iorio A . Policies on artificial intelligence chatbots among academic publishers: a cross-sectional audit. Res Integr Peer Rev. (2025) 10:1. doi: 10.1186/s41073-025-00158-y, 40022253 PMC11869395

[27] Cruz Rivera S Liu X Chan AW Denniston AK Calvert MJ SPIRIT-AI and CONSORT-AI Working Group . Guidelines for clinical trial protocols for interventions involving artificial intelligence: the SPIRIT-AI extension. Nat Med. (2020) 26:1351–63. doi: 10.1038/s41591-020-1037-7, 32908284 PMC7598944

[28] Liu X Cruz Rivera S Moher D Calvert MJ SPIRIT-AI and CONSORT-AI Working Group. Reporting guidelines for clinical trial reports for interventions involving artificial intelligence: the CONSORT-AI extension. Nat Med. (2020) 26:1364–74. doi: 10.1016/S2589-7500(20)30218-1 32908283 PMC7598943

[29] Ibrahim H Liu X Rivera SC Moher D Chan AW Sydes MR . Reporting guidelines for clinical trials of artificial intelligence interventions: the SPIRIT-AI and CONSORT-AI guidelines. Trials. (2021) 22:11. doi: 10.1186/s13063-020-04951-6, 33407780 PMC7788716

[30] Authorship and AI tools. COPE: Committee on Publication Ethics. Available online at: https://publicationethics.org/guidance/cope-position/authorship-and-ai-tools (Accessed November 18, 2025)

[31] Chatbots, generative AI, and scholarly manuscripts. Available online at: https://wame.org/page3.php?id=106 (Accessed November 18, 2025)

[32] Kocak Z. Publication ethics in the era of artificial intelligence. J Korean Med Sci. (2024) 39:e249. doi: 10.3346/jkms.2024.39.e249, 39189714 PMC11347185

[33] Topol EJ. High-performance medicine: the convergence of human and artificial intelligence. Nat Med. (2019) 25:44–56. doi: 10.1038/s41591-018-0300-7, 30617339

[34] Brinker TJ Hekler A Enk AH Klode J Hauschild A Berking C . Deep learning outperformed 136 of 157 dermatologists in a head-to-head dermoscopic melanoma image classification task. Eur J Cancer. (2019) 113:47–54. doi: 10.1016/j.ejca.2019.04.001, 30981091

[35] Rajkomar A Oren E Chen K Dai AM Hajaj N Hardt M . Scalable and accurate deep learning with electronic health records. NPJ Digit Med. (2018) 1:18. doi: 10.1038/s41746-018-0029-1, 31304302 PMC6550175

[36] Lundervold AS Lundervold A. An overview of deep learning in medical imaging focusing on MRI. Z Med Phys. (2019) 29:102–27. doi: 10.1016/j.zemedi.2018.11.002, 30553609

[37] Chappell M Edwards M Watkins D Marshall C Graziadio S. Machine learning for accelerating screening in evidence reviews. Cochrane Evid Synth Methods. (2023) 1:e12021. doi: 10.1002/cesm.12021, 40475071 PMC11795896

[38] Tsafnat G Glasziou P Choong MK Dunn A Galgani F Coiera E. Systematic review automation technologies. Syst Rev. (2014) 3:74. doi: 10.1186/2046-4053-3-74, 25005128 PMC4100748

[39] Fehr J Citro B Malpani R Lippert C Madai VI. A trustworthy AI reality-check: the lack of transparency of artificial intelligence products in healthcare. Front Digit Health. (2024) 6:1267290. doi: 10.3389/fdgth.2024.1267290, 38455991 PMC10919164

[40] AbuAlrob MA Mesraoua B. Harnessing artificial intelligence for the diagnosis and treatment of neurological emergencies: a comprehensive review of recent advances and future directions. Front Neurol. (2024) 15:1485799. doi: 10.3389/fneur.2024.1485799, 39463792 PMC11502371

[41] Flanagin A Kendall-Taylor J Bibbins-Domingo K. Guidance for authors, peer reviewers, and editors on use of AI, language models, and chatbots. JAMA. (2023) 330:702–3. doi: 10.1001/jama.2023.12500, 37498593

