# Supplementary material for: Artificial intelligence policies in neurology journals: a cross-sectional analysis
Source: Front Neurol. 2026 Feb 17;17:1766696. doi: 10.3389/fneur.2026.1766696 (PMC12953095; doi:10.3389/fneur.2026.1766696)
Supplement: Supplementary file 1 [file Supplementary_file_1.docx]

**Additional File 1. Biserial Correlation Analysis**

IF

Effect sizes were labelled following Funder's (2019) recommendations.

The Pearson's product-moment correlation between BiSerial_IF$`2023 Journal Impact Factor` and

BiSerial_IF$`AI tools allowed for Authorship` is positive, statistically not significant, and

tiny (r = 7.14e-04, 95% CI [-0.20, 0.20], t(98) = 7.07e-03, p = 0.994)

Effect sizes were labelled following Funder's (2019) recommendations.

The Pearson's product-moment correlation between BiSerial_IF$`2023 Journal Impact Factor` and

BiSerial_IF$`AI tools allowed in Manuscript Writing` is negative, statistically not

significant, and small (r = -0.18, 95% CI [-0.36, 0.02], t(98) = -1.78, p = 0.078)

Effect sizes were labelled following Funder's (2019) recommendations.

The Pearson's product-moment correlation between BiSerial_IF$`2023 Journal Impact Factor` and

BiSerial_IF$`AI tools allowed in content generation` is negative, statistically not

significant, and small (r = -0.14, 95% CI [-0.33, 0.06], t(98) = -1.38, p = 0.170)

Effect sizes were labelled following Funder's (2019) recommendations.

The Pearson's product-moment correlation between BiSerial_IF$`2023 Journal Impact Factor` and

BiSerial_IF$`AI tools allowed in image generation` is negative, statistically not

significant, and very small (r = -0.10, 95% CI [-0.29, 0.10], t(98) = -0.97, p = 0.332)

Effect sizes were labelled following Funder's (2019) recommendations.

The Pearson's product-moment correlation between BiSerial_IF$`2023 Journal Impact Factor` and

BiSerial_IF$`Require authors to disclose the use of AI during submission` is positive,

statistically not significant, and tiny (r = 7.14e-04, 95% CI [-0.20, 0.20], t(98) =

7.07e-03, p = 0.994)

SCImago Ranking

Effect sizes were labelled following Funder's (2019) recommendations.

The Pearson's product-moment correlation between BiSerial_SCI$`2023 SCImago Journal Rankings`

and BiSerial_SCI$`AI tools allowed for Authorship` is negative, statistically not

significant, and tiny (r = -0.01, 95% CI [-0.21, 0.18], t(98) = -0.14, p = 0.891)

Effect sizes were labelled following Funder's (2019) recommendations.

The Pearson's product-moment correlation between BiSerial_SCI$`2023 SCImago Journal Rankings`

and BiSerial_SCI$`AI tools allowed in Manuscript Writing` is negative, statistically not

significant, and small (r = -0.16, 95% CI [-0.34, 0.04], t(98) = -1.56, p = 0.121)

Effect sizes were labelled following Funder's (2019) recommendations.

The Pearson's product-moment correlation between BiSerial_SCI$`2023 SCImago Journal Rankings`

and BiSerial_SCI$`AI tools allowed in content generation` is negative, statistically not

significant, and small (r = -0.13, 95% CI [-0.32, 0.07], t(98) = -1.28, p = 0.203)

Effect sizes were labelled following Funder's (2019) recommendations.

The Pearson's product-moment correlation between BiSerial_SCI$`2023 SCImago Journal Rankings`

and BiSerial_SCI$`AI tools allowed in image generation` is negative, statistically not

significant, and small (r = -0.14, 95% CI [-0.33, 0.06], t(98) = -1.39, p = 0.168)

Effect sizes were labelled following Funder's (2019) recommendations.

The Pearson's product-moment correlation between BiSerial_SCI$`2023 SCImago Journal Rankings`

and BiSerial_SCI$`Require authors to disclose the use of AI during submission` is negative,

statistically not significant, and tiny (r = -0.01, 95% CI [-0.21, 0.18], t(98) = -0.14, p =

0.891)
